# Evaluation of groundwater and surface water quality and human risk assessment for trace metals in human settlements around the Bosomtwe Crater Lake in Ghana

**DOI:** 10.1186/s40064-016-3462-0

**Published:** 2016-10-18

**Authors:** Noah Kyame Asare-Donkor, Thomas Asare Boadu, Anthony Apeke Adimado

**Affiliations:** Department of Chemistry, Kwame Nkrumah University of Science and Technology, Kumasi, Ghana

**Keywords:** Health risk, Metal pollution, Surface water, Groundwater, Bosomtwe Crater Lake

## Abstract

Geogenic and anthropogenic activities introduce certain metals into the environment which tend to deteriorate the quality of both surface and groundwater in the Bosomtwe Crater Lake and its surroundings. In this study spatio-temporal variations in concentrations and risk assessment of selected trace metals (As, Fe, Pb, Zn, Cr, Cd and Ni) were investigated during the wet and dry seasons for surface and groundwater in selected human settlements around the lake. The levels of As, Cd and Ni were generally small and were below the detection limit of the instrument. The results showed no significant seasonal variations in the mean levels of Pb, Fe, Zn and Cr in water from the Bosomtwe Crater Lake. The hazard quotients and health hazard indices through ingestion and dermal contact of lake and groundwater in towns around the lake for both adults and children gave values which were below the acceptable limit of less than unity (< 1), indicating the absence of non-carcinogenic health risk to the communities. The study however reveals that ingestion of both lake and groundwater from the lake and its surroundings poses carcinogenic risk with regard to the level of Pb and Cr. Hence appropriate control measures and interventions should be put in place to protect the health of the human population in the study area.

## Background

Contamination of surface and groundwater by trace metals results in the deterioration of water quality which affect human health as well as the health of aquatic ecosystem (Krishna et al. 2009; Bataynen 2012). Trace metals in the aquatic environment creates an immense threat to the existence of organisms thriving in the area and the ecological integrity of the habitat, particularly as trace metals may enter the food chains, persist in the environment, bioaccumulate and bio-magnify. Though some trace metals in lower concentrations play important roles in metabolic processes of living organisms, high concentration have been observed to be toxic for human and aquatic life (Ouyang et al. 2002; Adepoju-Bello et al. 2009). High concentration of trace metals in water sources may lead to adverse effects such as deformities, cancer and bad health of aquatic animals and their terrestrial predators (Coeurdassier et al. 2010; Volpe et al. 2009; Kavacar et al. 2009). In humans trace metals above certain concentrations may lead to health problems including liver diseases, kidney problems and Geno toxic carcinogens (Knight et al. 1997; Gambrell 1994).

Metals enter rivers and lakes through a variety of sources such as eroded minerals within sediments, leaching of ore deposits, decomposing dead organic matter, fallout of atmospheric particulate and volcanism extruded products or anthropogenic sources including the discharge of liquid and solid waste, industrial or domestic effluents, channel and lake dredging etc. (Marcovecchio et al. 2007). Trace metals enter the human body through several routes such as food chain, direct ingestion, dermal contact, fume inhalation and particles through mouth and nose (Li and Zhang 2010; Wu et al. 2009; USEPA 1989, 2004).

For effective assessment of water quality it is important to identify potential human health effects of pollution in water. The traditional method for evaluating health effects directly compare the measured values with permissible limits, but it is not sufficiently reliable to provide detailed hazard levels and identify contaminants of the most concern. Health risk assessment is an important tool for estimating the potential health impact in aquatic ecosystems caused by various contaminants (Wu et al. 2010; Iqbal and Shah 2012). This method has been applied to evaluate the potential adverse health effects from exposure to contaminated water (Kavacar et al. 2009; Hartley et al. 1999; Sun et al. 2007). Although ingestion is considered the primary route of exposure to chemical contamination in drinking water sources inhalation and dermal absorption are increasingly being taken into account as important exposure pathways.

Lakes have important multi usage components including source of drinking water, irrigation, shipping, fishing, land scape entertainment and hydro-energy production (Yu et al. 2009). The Bosomtwe Crater lake which is a natural inland freshwater that originated from meteorite impact (Koeberl et al. 1997) and serve many functions including water for drinking and domestic use, fishing, transportation, tourism and landscape entertainment. Therefore safeguarding the quality of water in the lake and its surroundings is a great responsibility of the Government of Ghana, researchers and environmentalist for the conservation of this important water resource and world heritage site. However, there is limited information on the effects of the different anthropogenic activities on the water quality and the resultant health effects of the Bosomtwe Crater Lake. The aim of the study is to determine the levels of the selected metals (As, Cu, Fe, Cr, Cd and Pb) in the lake and groundwater and to evaluate the health risk associated with expose to these metals through oral ingestion and absorption through the skin. These metals were selected based on the dominant anthropogenic activities around the lake which include agriculture, proliferation of artisanal gold mining which contribute to soil pollution and land degradation, with the attendant exposure of the environment to trace metals pollution.

## Methods

### The study area

Lake Bosomtwe is centered at 06°32′N and 1°25′W and is one of the nineteen (19) confirmed impact structures known on earth and has an age of 1.07 million years. According to Koeberl et al. (1997) it is associated with one of the four tektites strewn fields in the Cote d’Ivoire tektite field. The lake is completely filled with water in a circular structure of roughly 8.5 km in diameter with a rim-to-rim diameter of about 10.5 km and has a depth of about 78–80 cm in its central part (Watkins 1994). The vegetation of the region around the lake is widely that of a dense tropical rainforest. The hydrogeology of the basin is dominated by aquifers of the crystalline basement rocks and the Birimian Province. Groundwater occurs mainly in the Birimian geological formations made up of the Lower Birimian (metasediment rocks) and the Upper Birimian (metavolcanic rocks). The Lower Birimian comprises of over 80 % of the total landmass of the basin while the Upper Birimian crops out in the eastern and extreme southern sections of the basin. Since the lake is a hydrological basin (Turner et al. 1995) all pollutants remain in the lake resulting in a complex and fragile ecosystem as they do not have self-cleaning ability and therefore readily accumulate pollutants (Lokeshwari and Chamdrappa 2006). The main occupation of the people around the lake is fishing and farming but quite recently there is a proliferation of artisanal gold activities around the area.

### Sampling and analysis

**Fig. 1:**
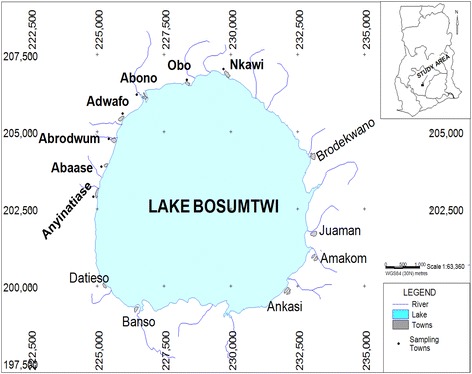
Map of study area showing towns where samples were taken in the Bosomtwe Crater Lake area

Water samples were collected from the lake and bore holes from seven human settlements around the Bosomtwe Crater Lake namely at Anyinatiase, Nkowi, Adwafo, Abrodwum, Obo, Abono and Abease as indicated in Fig. 1. Sampling was done every 3 months from November, 2012 to August, 2013.

Samples were collected into sterile screw capped plastic containers which had been washed with detergents, 4.0 mol dm^−3^ nitric acid solution and distilled water and dried in an oven. The pH, conductivity total dissolved solids and temperature were measured on the site using CyberScan PC 650 multimeter whilst turbidity was measured with a Hanna HI 93414 turbidity meter. The samples were then filtered through pre-washed 0.45 μm Millipore nitrocellulose filters to remove any suspensions, acidified with 6 M Analar HNO_3_ (2 ml l^−1^) to keep pH < 2 (USEPA 2003), transported in an ice chest to the laboratory and stored at −4 °C in a refrigerator. The trace metals were determined using Atomic Absorption Spectrophotometer (Model; Varian 220) using air-acetylene flame at a temperature of about 2300 °C. The appropriate hollow cathode lamps (HCL) for each of the elements As, Fe, Pb, Zn, Cr, Cd and Ni were employed as common radiation source at operating wavelengths of 193.7, 248.3, 217.0, 213.9, 357.9, 228.8 and 232.0 nm respectively.

### Quality control

Quality control measures for the analysis were implemented through the analytical protocols, including sampling and sample preservation, instrument performance evaluation, calibration of instrument, recovery and reagent blank and replicate analyses. The instrument performance evaluation involved optimizing instrument parameters followed by sensitivity check.

### Statistical analysis

The results were analyzed statistically employing Microsoft Excel (2010 edition) and statistical Package for Social Science (IBM SPSS version 20). Multivariate statistics in terms of principal component analysis (PCA) cluster analysis were carried out using the varimax-normalized rotation on the data (Shah et al. 2012; Iqbal and Shah 2012).

### Health risk assessment methodologies

The human health risk assessment methodologies for aquatic ecosystems has been described literature (Li and Zhang 2010; USEPA 1989, 2005; Wu et al. 2009). The ingestion and dermal absorption are common for water exposure (USEPA 1989, 2005; Wu et al. 2009). The numeric expressions for risk assessment as obtained from the USEPA Risk Assessment Guidance for Superfund (RAGS) methodology (USEPA 1989) are given as follows:

1 $$D_{\text{ing}} = \frac{Cwater \times IR \times EF \times ED}{BW \times AT}$$

2 $$D_{\text{derm}} = \frac{Cwater \times SA \times KP \times ET \times EF \times ED \times CF}{BW \times AT}$$

where *D*_ing_ is exposure dose through ingestion of water (μg/kg/day); *D*_derm_ is exposure dose through dermal absorption (μg/kg/day); *C* water is concentration of the estimated metals in water (μg/L); IR is ingestion rate (2.2 L/day for adults; 1.8 L/day for children); EF is exposure frequency (350 days/year); ED is exposure duration (70 years for adults; and 6 years for children); BW is average body weight (70 kg for adults; 15 kg for children); AT is averaging time (25,550 days for adults; 2190 days for children); SA is exposed skin area (18,000 cm^2^ for adults; 6600 cm^2^ for children); ET is exposure time (0.58 h/day for adults; 1 h/day for children); CF is unit conversion factor (0.001 L/cm^3^); and *Kp* is dermal permeability coefficient (cm/h). The dermal permeability coefficient for Fe, Pb, Zn, Cr and Ni are given as 1.0 × 10^−3^, 4.0 × 10^−3^, 6.0 × 10^−3^, 2.0 × 10^−3^ and 4.0 × 10^−3^ cm/h respectively (USEPA 1989, 2005; Wu et al. 2009; Liang et al. 2011).

Potential non-carcinogenic risks for exposure to contaminants were determined by comparing the calculated contaminant exposures from each exposure route with the reference dose (RfD) (USEPA 1989). The hazard quotient (HQ) which is a numeric estimate of the systemic toxicity potential posed by a single element within a single route of exposure was calculated using the relation:

3 $$HQ_{\text{ing/derm}} = \frac{{D_{ing/derm} }}{{RfD_{ing/derm} }}$$

where HQ_ing/derm_ is hazard quotient via ingestion or dermal contact and Rf*D*_ing/derm_ is oral/dermal reference dose (μg/kg/day). The Rf*D*_ing_ and Rf*D*_derm_ values were obtained from the literature (Li and Zhang 2010; USEPA 1989; Wu et al. 2009; Liang et al. 2011).

The overall potential for non-carcinogenic effects posed by more than one element was evaluated by integrating the computed HQs for each element and expressed as a hazard index (HI) (USEPA 1989):

4 $${\text{HI}} = \mathop \sum \limits_{i = 1}^{n} HQ_{ing/derm}$$

where HI_ing/derm_ is hazard index via ingestion or dermal contact. Chronic daily intake (CDI) was calculated using the relation:

5 $${\text{CDI}}_{\text{ing}} = {\text{C}}_{\text{water}} \times \frac{DI}{BW}$$

where C_water_, DI and BW represent the concentration of trace metal in water in (µg/kg), average daily intake of water and body weight respectively.

Cancer risk (CR) was calculated using the formula:

6 $${\text{CR}}_{\text{ing}} = \frac{{D_{ing} }}{{SF_{ing} }}$$

where SF_ing_ is the cancer slop factor. The SF_ing_ for Pb is 8.5 and Cr is 5.0 × 10^2^ mg/kg/day (USEPA 1989, 2005; Yu et al. 2010; Vieira et al. 2011).

## Results and discussion

Table 1 shows the mean pH, temperature and conductivity of water samples from Lake Bosomtwe and Groundwater in some towns around the lake in the wet and dry seasons. Table 2 also show the mean concentrations of As, Fe, Pb, Zn, Cr, Cd and Ni in water samples from Lake Bosomtwe and Groundwater in some towns around the lake in the wet and dry seasons. The values show that the water from the lake in the dry season is alkaline with the pH values above the recommended WHO standards of 6.50–8.50. However, the pH for lake water in the wet season and those of the groundwater for both dry and wet seasons were observed to be within the WHO range. These values indicate that there was not much difference in the pH in both the water from the lake and groundwater in the surroundings. Thus there in no significant variability of pH due to seasonal contamination. Almost all the water from the lake has the average temperature of 30 °C and the water from the groundwater has the average temperature of 29 °C. Generally the mean conductivities were high indicating that the water contains large amounts of ions which were responsible for such conductivities (APHA 2005: Jain et al. 2005). The health effects for consuming water with high conductivity are the disturbances of salt and water balance which have adverse effects on some myocardial patients and individuals with high blood pressure (Fatoki and Awofulu 2003). The total dissolved solids which may be a measure of the dissolution mechanism of inorganic and organic materials in water for both groundwater and lake water were generally low and below the WHO value of 1000 mg/L for both seasons. Turbidity was also low for both sources of water for both dry and wet seasons and were also below the WHO limit of 5 NTU. Turbidity in water is caused by colloidal matters or suspended particles that obstruct light transmission through the water may originate from the presence of inorganic or organic matter or the combination of both.

**Table 1:** Mean values of some physical parameters in lake and groundwater samples collected from seven human settlements along the Bosomtwe Crater Lake in dry and wet seasons

Season	Settlement	Lake water					Groundwater				
		pH	Temperature (°C)	TDS (mg/L)	Turbidity/NTU	Conductivity (µs cm^−1^)	pH	Temperature (°C)	TDS (mg/L)	Turbidity/NTU	Conductivity (µs cm^−1)^
Dry (*N = 105)	Anyinatiase	9.83 ± 0.01	30.00 ± 0.00	237.40 ± 5.46	1.32 ± 0.03	1263.40 ± 8.63	6.77 ± 0.27	29.00 ± 0.00	197.99 ± 7.01	1.00 ± 0.01	1375.60 ± 177.91
	Nkowi	9.77 ± 0.01	30.00 ± 0.00	215.25 ± 3.58	0.71 ± 0.01	1307.30 ± 73.11	7.19 ± 0.30	31.00 ± 1.41	178.79 ± 3.21	0.97 ± 0.01	1277.10 ± 3.96
	Adwafo	9.82 ± 0.02	30.00 ± 0.00	215.80 ± 3.45	0.87 ± 0.04	1201.10 ± 41.44	7.18 ± 0.14	29.00 ± 1.41	200.46 ± 2.32	0.80 ± 0.03	1395.00 ± 26.09
	Abrodwum	9.75 ± 0.01	30.00 ± 0.00	206.03 ± 4.68	1.06. ± 0.02	1250.45 ± 8.27	7.42 ± 0.16	28.00 ± 0.00	198.77 ± 4.33	0.71 ± 0.02	1242.35 ± 9.69
	Obo	9.71 ± 0.01	29.00 ± 0.00	197.95 ± 5.21	0.90 ± 0.02	1787.05 ± 172.75	7.14 ± 0.04	30.00 ± 0.00	189.33 ± 1.24	0.67 ± 0.01	1285.28 ± 0.95
	Abono	8.98 ± 0.00	28.50 ± 0.00	208.10 ± 4.28	0.88. ± 0.04	1266.23 ± 6.75	7.19 ± 0.09	30.00 ± 0.00	199.87 ± 2.45	0.42 ± 0.01	1264.60 ± 5.37
	Abease	9.82 ± 0.02	30.00 ± 0.00	197.95 ± 3.32	1.00 ± 0.02	1197.35 ± 60.60	7.34 ± 0.23	29.50 ± 0.71	200.06 ± 3.42	0.50 ± 0.00	1238.63 ± 24.71
Wet (*N = 105)	Anyinatiase	6.88 ± 0.11	29.00 ± 0.00	206.70 ± 4.11	0.90 ± 0.02	1241.85 ± 6.72	6.77 ± 0.27	29.00 ± 0.00	256.87 ± 5.58	0.90. ± 0.02	1356.1 ± 150.40
	Nkowi	6.99 ± 0.01	31.50 ± 0.71	199 67 ± 3.24	1.64 ± 0.01	1264.90 ± 37.90	7.19 ± 0.30	31.00 ± 1.41	211.43 ± 3.42	1.10 ± 0.03	1277.05 ± 3.89
	Adwafo	7.14 ± 0.08	29.50 ± 0.71	180.14 ± 4.38	0.95 ± 0.03	1212.65 ± 24.82	7.18 ± 0.14	29.00 ± 1.41	209.76 ± 5.62	0.90 ± 0.01	1392.38 ± 24.25
	Abrodwum	7.48 ± 0.08	28.00 ± 0.00	200.84 ± 3.68	1.23 ± 0.03	1260.00 ± 10.89	7.42 ± 0.16	28.00 ± 0.00	198.77 ± 4.18	0.81 ± 0.01	1218.35 ± 24.25
	Obo	7.13 ± 0.03	30.00 ± 0.00	189.23 ± 5.13	1.32 ± 0.04	1269.75 ± 0.78	7.14 ± 0.04	30.00 ± 0.00	202.33 ± 3.01	0.78 ± 0.01	1282.30 ± 3.25
	Abono	7.16 ± 0.06	30.00 ± 0.00	217.54 ± 3.56	0.98 ± 0.01	1243.50 ± 12.73	7.19 ± 0.09	30.00 ± 0.00	199.87 ± 4.32	0.52 ± 0.00	1260.55 ± 0.35
	Abease	7.25 ± 0.11	29.75 ± 0.35	170.44 ± 2.31	1.02 ± 0.03	1258.15 ± 0.92	7.34 ± 0.23	29.50 ± 0.71	188.96 ± 2.34	0.61 ± 0.01	1238.30 ± 25.17

* N = sample size, i.e. 5 samples each for groundwater and lake water for each of the 7 settlements for 3 months within each season

**Table 2:** Mean concentrations in mg/L of some trace metals in water samples collected from seven human settlements along the Bosomtwe Crater Lake in the dry and wet seasons

Season	Settlement	Lake water							Groundwater						
		As	Fe	Pb	Zn	Cr	Cd	Ni	As	Fe	Pb	Zn	Cr	Cd	Ni
Dry (*N = 105)	Anyinatiase	<0.01	0.20 ± 0.02	0.17 ± 0.01	0.12 ± 0.01	0.03 ± 0.00	<0.01	<0.01	<0.01	0.57 ± 0.41	0.12 ± 0.00	0.14 ± 0.00	0.01 ± 0.00	<0.01	<0.01
	Nkowi	<0.01	0.37 ± 0.08	0.06 ± 0.07	0.21 ± 0.00	0.01 ± 0.00	<0.01	<0.01	<0.01	0.28 ± 0.04	0.10 ± 0.01	0.28 ± 0.00	0.02 ± 0.00	<0.01	<0.01
	Adwafo	<0.01	0.22 ± 0.02	0.13 ± 0.01	0.10 ± 0.00	<0.01	<0.01	<0.01	<0.01	0.58 ± 0.08	0.17 ± 0.01	0.25 ± 0.00	0.03 ± 0.01	<0.01	<0.01
	Abrodwum	<0.01	0.28 ± 0.04	0.14 ± 0.03	0.14 ± 0.00	0.05 ± 0.00	<0.01	<0.01	<0.01	0.20 ± 0.01	0.16 ± 0.02	0.13 ± 0.00	0.02 ± 0.00	<0.01	<0.01
	Obo	<0.01	0.38 ± 0.05	0.07 ± 0.00	0.21 ± 0.01	<0.01	<0.01	<0.01	<0.01	0.42 ± 0.04	0.10 ± 0.01	0.20 ± 0.00	0.02 ± 0.00	<0.01	<0.01
	Abono	<0.01	0.35 ± 0.04	0.09 ± 0.01	0.13 ± 0.01	0.02 ± 0.00	<0.01	0.02 ± 0.00	<0.01	0.29 ± 0.05	0.09 ± 0.01	0.19 ± 0.01	0.03 ± 0.00	<0.01	0.02 ± 0.00
	Abease	<0.01	0.27 ± 0.02	0.16 ± 0.02	0.12 ± 0.00	0.02 ± 0.00	<0.01	0.02 ± 0.00	<0.01	0.21 ± 0.01	0.16 ± 0.03	0.12 ± 0.01	0.02 ± 0.00	<0.01	0.02 ± 0.00
Wet (*N = 105)	Anyinatiase	<0.01	0.27 ± 0.12	0.11 ± 0.03	0.18 ± 0.03	0.02 ± 0.01	<0.01	<0.01	<0.01	1.02 ± 1.29	0.12 ± 0.00	0.15 ± 0.00	0.02 ± 0.00	<0.01	<0.01
	Nkowi	<0.01	0.29 ± 0.06	0.10 ± 0.00	0.12 ± 0.05	0.01 ± 0.00	<0.01	<0.01	<0.01	0.39 ± 0.07	0.09 ± 0.01	0.28 ± 0.00	0.01 ± 0.01	<0.01	<0.01
	Adwafo	<0.01	0.28 ± 0.03	0.09 ± 0.02	0.11 ± 0.00	0.02 ± 0.00	<0.01	<0.01	<0.01	0.53 ± 0.05	0.07 ± 0.09	0.25 ± 0.01	0.03 ± 0.02	<0.01	<0.01
	Abrodwum	<0.01	0.37 ± 0.05	0.05 ± 0.05	0.14 ± 0.00	0.02 ± 0.00	<0.01	<0.01	<0.01	0.32 ± 0.06	0.07 ± 0.08	0.14 ± 0.01	0.02 ± 0.00	<0.01	<0.01
	Obo	<0.01	1.99 ± 0.77	0.06 ± 0.00	0.16 ± 0.03	0.01	<0.01	<0.01	<0.01	0.46 ± 0.05	0.07 ± 0.02	0.15 ± 0.03	0.01 ± 0.01	<0.01	<0.01
	Abono	<0.01	0.49 ± 0.08	0.04 ± 0.02	0.12 ± 0.01	0.02 ± 0.00	<0.01	0.02	<0.01	0.37 ± 0.05	0.05 ± 0.02	0.22 ± 0.01	0.02 ± 0.00	<0.01	0.19 ± 0.05
	Abease	<0.01	0.30 ± 0.02	0.07 ± 0.08	0.09 ± 0.01	0.02 ± 0.00	<0.01	0.02	<0.01	0.26 ± 0.03	0.06 ± 0.07	0.16 ± 0.02	0.01 ± 0.00	<0.01	0.19 ± 0.03

* N = sample size, i.e. 5 samples each for groundwater and lake water for each of the 7 settlements for 3 months within each season

### Trace metals

The mean levels of trace metals showed generally no significant differences between lake and groundwater or during the dry and wet seasons (Table 2). The levels of the metals arsenic and cadmium, were generally small and generally below the detection limit of the instrument, Varian AAS 220 used which is 0.01 mg/L. This shows that their levels may be below those of WHO standard values of 0.01 mg/L for As, 0.003 mg/L for Cd and 0.02 mg/L for Ni.

Fe recorded mean levels in the range of 0.20 ± 0.02–0.38 ± 0.05 mg/L and 0.20 ± 0.01–0.58 ± 0.08 mg/L in lake water and groundwater respectively in the dry season for all the sampling stations. In the wet season the mean values recorded for Fe were in the range of 0.27 ± 0.12–1.99 ± 0.77 mg/L and 0.26 ± 0.03–1.02 ± 1.02 mg/L for lake water and groundwater respectively. These give rise to overall mean values for both lake and groundwater around Lake Bosomtwe exceeding the WHO standard value of 0.3 mg/L (1993). Large amount of ingested iron may results in high iron levels in the blood which lead to reaction between iron and peroxides to produce free radicals. These free radicals are highly reactive and can damage DNA, protein lipids and other cellular components. Iron typically damage cells in the heart, liver and elsewhere which can cause significant adverse effect including coma, metabolic acidosis, shock, liver failure, coagulopathy, adult respiratory distress syndrome, long-term organ damage and even death (Dina et al. 2006; Alada, 2000; Ferner 2001).

Pb recorded mean levels in the range of 0.06 ± 0.07–0.17 ± 0.01 mg/L and 0.09 ± 0.01–0.17 ± 0.00 mg/L in lake water and groundwater respectively in the dry season for all the sampling stations. In the wet season the mean values recorded for Pb were in the range of 0.04 ± 0.02–0.11 ± 0.03 mg/L and 0.06 ± 0.07–0.12 ± 0.00 mg/L for lake water and groundwater respectively. All the recorded mean levels for Pb were above the WHO recommended standard of 0.01 mg/L. Mean Pb levels were generally relatively higher in the wet season than the dry season both lake and groundwater (Table 2). Lead damages the nervous connections especially in young children and cause blood and brain disorders.

Lead poisoning typically results from ingestion of food and water contaminated with lead. Several studies have shown that chronic lead exposure reduces nerve conduction velocity in peripheral nerves in adult subjects without clinical symptom or sign of disease (Skerfring 1993). Long-term exposure to lead cause small increase in blood pressure especially middle-aged and older people and can cause anaemia. Exposure to high level can severely damage the brain and kidney in males (Golub 2005).

Zn recorded mean levels in the range of 0.10 ± 0.00 to 0.21 ± 0.00 mg/L and 0.12 ± 0.01–0.28 ± 0.00 mg/L in lake water and groundwater respectively in the dry season for all the sampling stations. In the wet season the mean values recorded for Zn were in the range of 0.09 ± 0.01–0.18 ± 0.03 mg/L and 0.14 ± 0.01–0.28 ± 0.00 mg/L for lake water and groundwater respectively. In general the levels of Zn in the water samples from groundwater were higher than those in the water samples from the lake. Zn levels at Nkowi and Obo were similarly higher in the wet season as compared to the dry season. Zinc is an essential mineral of exceptional biological and public health importance (Hambridge and Krebs 2007). Zinc is believed to possess antioxidant properties, which may protect human beings against accelerated aging of the skin and muscles in the body. It also helps to speed up the healing process after injury (Milbury and Richer 2008). Zinc toxicity can occur in both acute and chronic forms. Acute adverse effects of high intake of zinc include nausea, vomiting, loss of appetite, abdominal cramps diarrhea and headache (Prasad 2005). Excessive amount of zinc is harmful as it suppresses copper and iron absorption (Fosmire 1990). Zinc deficiency is associated with chronic liver disease, chronic renal disease, sickle cell disease, diabetes, malignancy and other chronic illness (Prasad 2005).

Cr recorded mean levels in the range of below detection to 0.05 ± 0.00 mg/L and 0.02 ± 0.00 to 0.03 ± 0.00 mg/L in lake water and groundwater respectively in the dry season for all the sampling stations. In the wet season the mean values recorded for Cr were in the range of below detection to 0.02 ± 0.00 mg/L and 0.01 ± 0.00 to 0.03 ± 0.02 mg/L for lake water and groundwater respectively. Chromium has no verified biological role and has been classified as not essential for mammals (Bona et al. 2010). Cr(VI) is very toxic and mutagenic but has not been established as a carcinogen when in solution although it may cause allergic contact dermatitis (ACD) (Bona et al. 2010). Several in vitro studies indicated that high concentration of Cr(III) in the cell can lead to DNA damage (Eastmond et al. 2008).

The level of Ni were generally below the detection limit of the instrument nickel except at two sampling stations where they recorded levels of 0.19 ± 0.05 mg/L and 0.19 ± 0.03 mg/L. Nickel plays an important role in the biology of the micro-organisms and plants (Astrid et al. 2007). However, high concentrations of nickel have been reported to be toxic and have carcinogenic effects for a wide range of life forms (ATSDR 2000; Goyer 1996; IARC 1990). Sensitized individuals may show allergy to nickel resulting in skins-dermatitis (skin itch). Once a person is sensitized to nickel, any further contact will produce a reaction and adverse health effects can occur at far lower concentrations compared to non-sensitized individuals (ATSDR 2000).

### Multivariate analysis

**Fig. 2:**
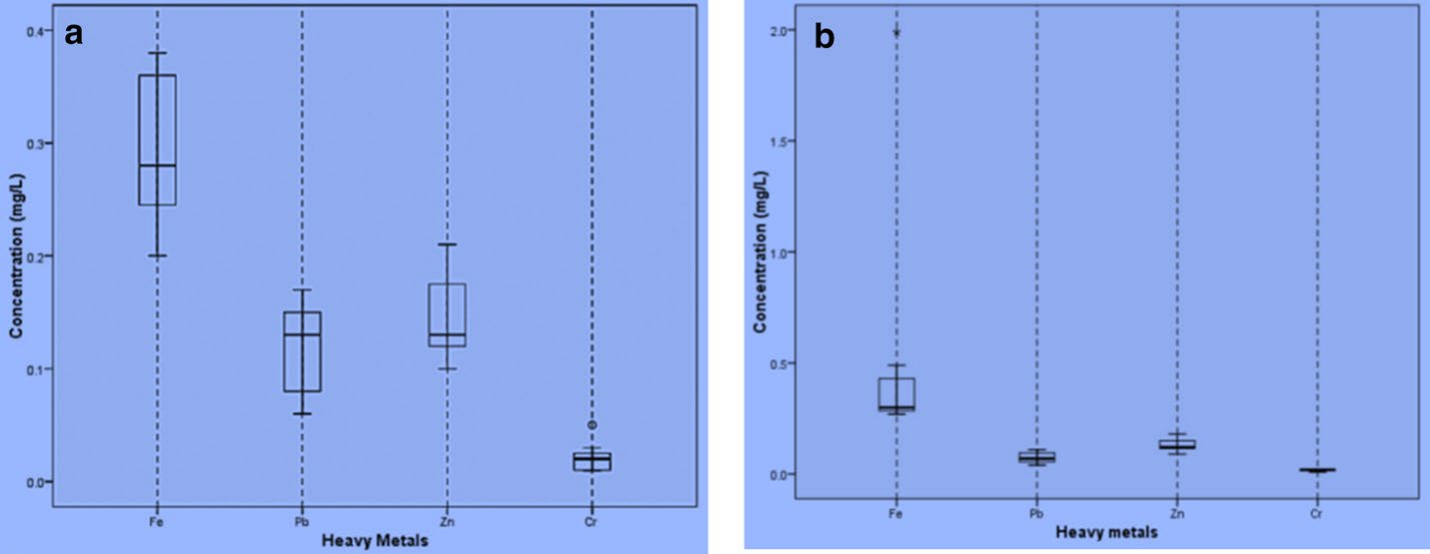
Box and whisker plots of metals in the water samples from the Bosomtwe Crater Lake in the **a** dry and **b** wet seasons

**Fig. 3:**
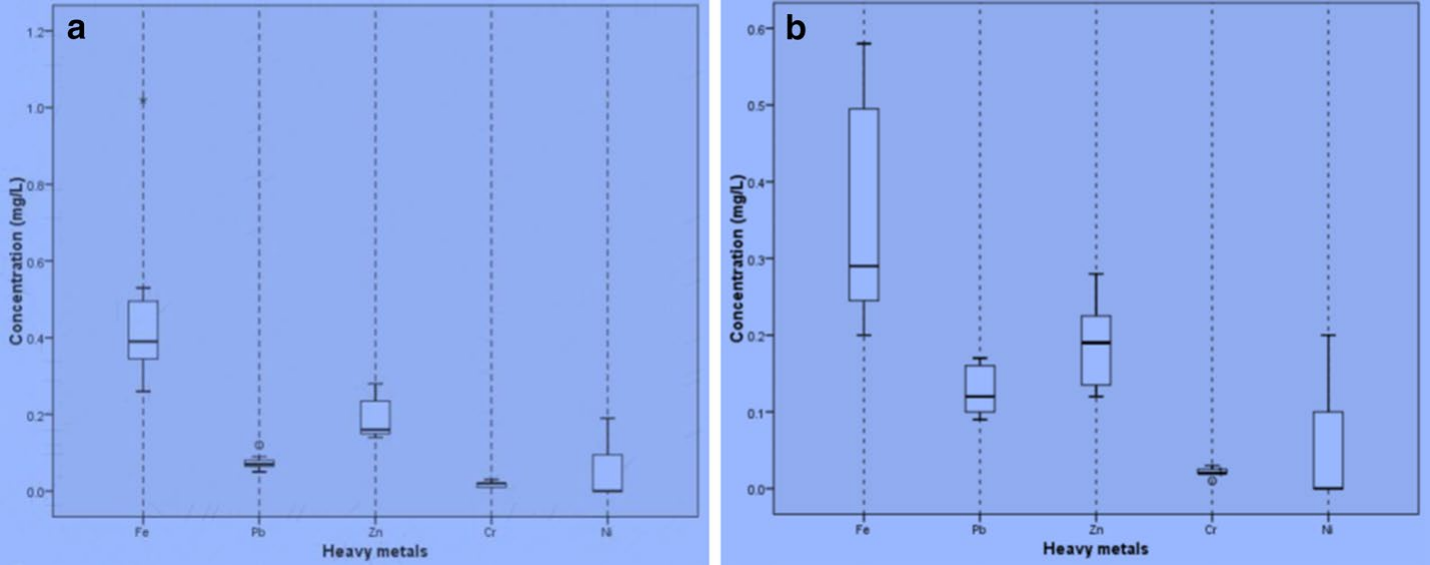
Box and whisker plots of metals in the groundwater sample from the Bosomtwe Crater Lake in the **a** dry and **b** wet seasons

Figures 2 and 3 show the Box and Whisker plots of metals in the surface and groundwater from the Bosomtwi Crater Lake and its surroundings in the dry and wet seasons. The asterisks and white circles represent outliers. The effects of outliers on the outcome of the multivariate analysis were not significant at 95 % confidence limit by Student’s t test. Tables 3 and 4 show the Principal component/factor analysis (PCA/FA) for selected trace metals in lake water and groundwater respectively for wet and dry seasons. PCA/FA identified two principal components (PC) for lake water in both wet and dry seasons with % total variance of 80.99 and 72.73 respectively. PCA/FA for groundwater identified two principal components (PC) for the wet season with % total variance of 73.80 and three principal components for the dry season with % total variance of 87.33. The ratios calculated for these trace metals suggest mineral weathering as a vital geochemical process that control the concentrations of the trace metals in both the lake water and groundwater in the study area.

**Table 3:** Factor loading for selected trace metals in lake water samples for wet and dry seasons

Variable	Dry season		Wet season	
	PC 1	PC 2	PC 1	PC 2
Fe	−0.541	0.236	0.338	0.677
Pb	0.564	−0.074	0.372	−0.611
Zn	−0.531	−0.262	0.539	0.059
Cr	0.318	−0.139	−0.406	−0.292
Ni	0.080	0.922	−0.540	0.282
Eigenvalues	2.918	1.131	2.234	1.403
% of variance	58.360	22.627	44.677	28.052
Cumulative %	58.360	80.987	44.677	72.728

**Table 4:** Factor loading for selected trace metals in groundwater samples for wet and dry seasons

Variable	Dry season			Wet season	
	PC 1	PC 2	PC 3	PC 1	PC 2
Fe	0.492	−0.366	0.142	0.577	−0.005
Pb	−0.144	−0.069	0.913	0.625	−0.083
Zn	0.635	0.285	−0.167	−0.125	0.643
Cr	0.366	0.672	0.329	0.000	0.713
Ni	−0.447	0.574	−0.104	−0.511	−0.265
Eigenvalues	1.935	1.312	1.120	2.300	1.390
% of variance	38.701	26.232	22.395	45.998	27.801
Cumulative %	38.701	64.933	87.328	45.998	73.799

**Fig. 4:**
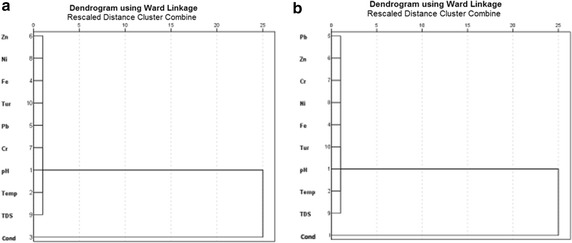
Dendrogram of trace metals pH, conductivity and temperature in the lake water samples from Bosomtwe Crater Lake in the **a** dry and **b** wet seasons using ward’s method

**Fig. 5:**
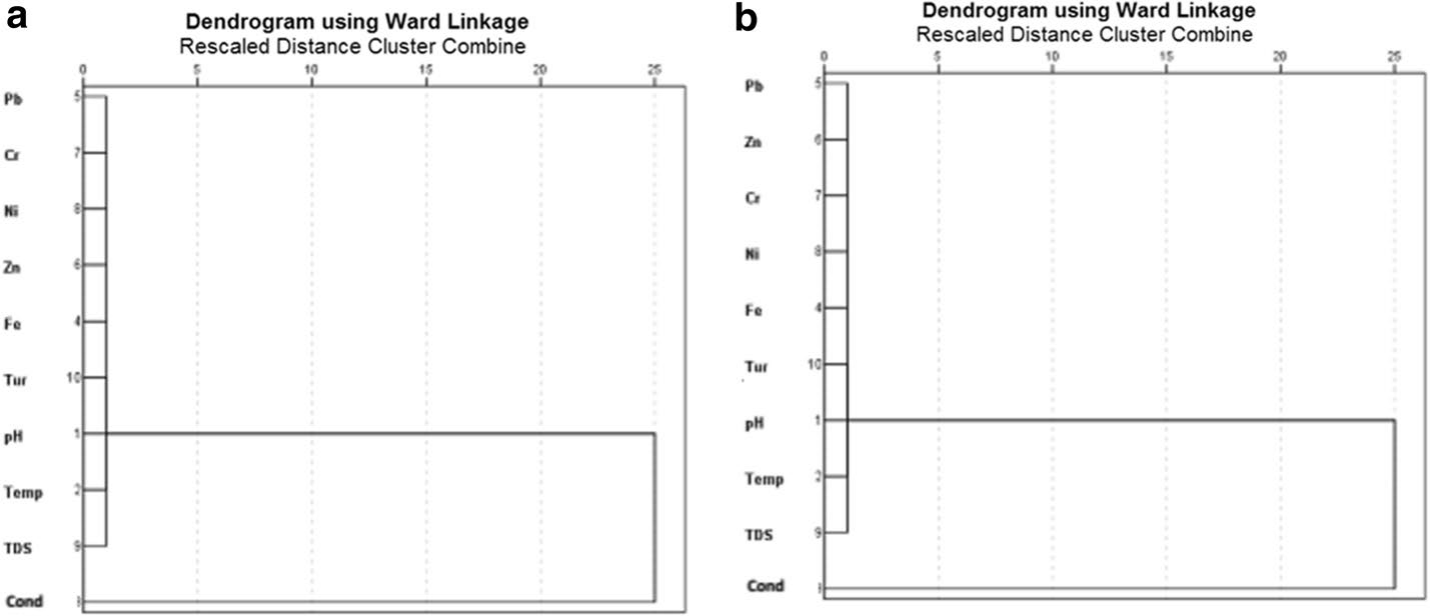
Dendrogram of trace metals pH, conductivity and temperature in groundwater samples from the surroundings of Bosomtwe Crater Lake in the **a** dry and **b** wet seasons using ward’s method

The R-mode HCA was used to determine the relationship among the various trace metals, pH, conductivity and temperature using Ward’s method (Squared Euclidean distance as measure of similarity). Cluster analysis (CA) grouped these parameters into clusters on the basis of similarities within a group and dissimilarities between different groups. Parameters belonging to the same cluster are likely to have originated from a common source. The R-mode CA produced two clusters based on spatial similarities and dissimilarities (Figs. 4, 5) for the lake and groundwater for both dry and wet seasons. Cluster 1 for dry seasons for the groundwater and lake water contained the same parameters (i.e. Fe, Pb, Zn, Cr, Ni, TDS, Turbidity and Temp) whereas cluster 2 contained only pH and conductivity. Similarly for the wet seasons Cluster 1 for both groundwater and lake water contained the same parameters (Fe, Pb, Zn, Cr, Ni, TDS, Turbidity and Temp) whereas cluster 2 contained pH and conductivity. The fact that cluster 1 for both lake water and groundwater for both seasons contained Pb, Zn, Cr, Ni and Fe seem to suggest that these metals are mainly of lithogenic rather than anthropogenic sources.

### Health risk assessment for some selected metals

The results of non-carcinogenic health risk assessment for the selected metals in both lake and groundwater for adults and children via ingestion and dermal routes are given in Tables 5 and 6. Human beings are exposed to trace metals through direct ingestion, inhalation through mouth and nose, and dermal absorption through skin. The health risk associated with ingestion of water depends on the volume of water consumed and the weight of the individual. Hence health risk assessment was determined using the maximum and minimum concentration of the Fe, Pb, Zn, Cr and Ni in the lake and groundwater. The results indicate that the hazard quotient through injection of water HQ_ing_ from the Bosomtwe Crater Lake for all the metals during both wet and dry seasons were less than unity for both adults and children which indicates that these metals could pose minimum hazard to local residents. The HQ_derm_ values were also found to be less than unity which means that dermal adsorption of the metals may have little or no health threat. Health hazard indices (HI) on exposure to water from both lake water and ground water through ingestion and dermal contacts for both seasons are less than unity. It is therefore obvious from the results that in both cases the observed values are below the safe limit of unity which clearly indicates that there was no cumulative potential of adverse risk in water sampled via direct ingestion or dermal ingestion to the Inhabitants. The chronic risk assessment (CDI) for the metals in the groundwater samples from habitats surrounding the Bosomtwe Crater Lake and water samples from the lake through the ingestion pathway is given in Table 7. Generally health risk assessment indices like HQ and CDI and the overall non-carcinogenic health risk assessment (HI) less than unity are indicative of less significant risk through the ingestion route or dermal contact (Moore and Ramamoorthy 1984; Wu et al. 2009).

**Table 5:** Health risk assessment for the metals in the lake water samples from the Bosomtwe Crater Lake through ingestion and dermal absorption pathways during the wet and dry seasons for adults and children

	RfD_ing_ (µg/kg/day)	RfD_derm_ (µg/kg/day)	Wet season				Dry season			
			Adults		Children		Adults		Children	
			HQ_ing_	HQ_derm_	HQ_ing_	HQ_derm_	HQ_ing_	HQ_derm_	HQ_ing_	HQ_derm_
Fe	700	140	1.16 × 10^−5^–8.57 × 10^−5^	2.76 × 10^−7^–2.03 × 10^−6^	4.44 × 10^−5^–3.27 × 10^−4^	8.14 × 10^−7^–6 × 10^−6^	9.47 × 10^−5^–1.64 × 10^−5^	2.25 × 10^−7^–3.88 × 10^−7^	5.75 × 10^−3^–1.40 × 10^−2^	6.63 × 10^−7^–1.15 × 10^−6^
Pb	1.4	0.42	8.61 × 10^−4^–2.37 × 10^−3^	5.45 × 10^−5^–1.5 × 10^−4^	3.29 × 10^−3^–9.04 × 10^−3^	1.61 × 10^−4^–4.42 × 10^−4^	1.51 × 10^−3^–3.66 × 10^−3^	9.53 × 10^−5^–2.32 × 10^−4^	3.62 × 10^−5^–6.25 × 10^−5^	2.81 × 10^−4^–6.83 × 10^−4^
Zn	300	120	9.04 × 10^−6^–1.81 × 10^−5^	6.44 × 10^−7^–1.29 × 10^−6^	3.45 × 10^−5^–6.9 × 10^−5^	1.9 × 10^−6^–3.8 × 10^−6^	1 × 10^−5^–2.11 × 10^−5^	7.15 × 10^−7^–1.5 × 10^−6^	3.84 × 10^−5^–8.05 × 10^−5^	2.11 × 10^−6^–4.43 × 10^−6^
Cr	3	0.075	0–2.01 × 10^−4^	0–7.63 × 10^−5^	0–7.67 × 10^−4^	0–2.25 × 10^−4^	0–5.02 × 10^−4^	0–1.91 × 10^−4^	0–1.92 × 10^−3^	0–5.63 × 10^−4^
Ni	20	5.4	0–3.01 × 10^−5^	0–2.12 × 10^−6^	0–1.15 × 10^−4^	0–6.25 × 10^−6^	0–3.01 × 10^−5^	0–2.12 × 10^−6^	0–1.15 × 10^−4^	0–6.25 × 10^−6^
HI_ing/derm_	–	–	8.82 × 10^−4^–2.70 × 10^−3^	5.54 × 10^−5^–2.32 × 10^−4^	3.37 × 10^−3^–1.03 × 10^−2^	1.64 × 10^−4^–6.83 × 10^−4^	1.53 × 10^−3^–4.23 × 10^−3^	9.63 × 10^−5^–4.27 × 10^−3^	5.83 × 10^−3^–1.62 × 10^−2^	2.84 × 10^−4^–1.95 × 10^−3^

**Table 6:** Health risk assessment for the metals in the groundwater samples from groundwater in settlements around the Bosomtwe Crater Lake through ingestion and dermal absorption pathways during the wet and dry seasons for adults and children

	RfD_ing_ (µg/kg/day)	RfD_derm_ (µg/kg/day)	Wet season				Dry season			
			Adults		Children		Adults		Children	
			HQ_ing_	HQ_derm_	HQ_ing_	HQ_derm_	HQ_ing_	HQ_derm_	HQ_ing_	HQ_derm_
Fe	700	140	1.2 × 10^−5^–4.39 × 10^−5^	2.66 × 10^−7^–1.04 × 10^−6^	4.27 × 10^−5^–1.68 × 10^−4^	7.84 × 10^−7^–3.07 × 10^−6^	8.61 × 10^−6^–2.5 × 10^−5^	2.04 × 10^−7^–5.92 × 10^−7^	3.29 × 10^−5^–9.53 × 10^−5^	6.03 × 10^−7^–1.75 × 10^−6^
Pb	1.4	0.42	1.08 × 10^−4^–2.58 × 10^−3^	6.81 × 10^−5^–1.63 × 10^−4^	4.11 × 10^−3^–9.86 × 10^3^	2.01 × 10^−4^–4.82 × 10^−4^	2.94 × 10^−3^–3.66 × 10^−3^	1.23 × 10^−4^–2.32 × 10^−4^	7.40 × 10^−3^–1.40 × 10^−2^	3.62 × 10^−4^–6.83 × 10^−4^
Zn	300	120	1.41 × 10^−5^–2.81 × 10^−5^	1 × 10^−6^–2 × 10^−6^	5.37 × 10^−5^–1.07 × 10^−4^	2.95 × 10^−6^–5.91 × 10^−6^	1.31 × 10^−5^–2.81 × 10^−5^	9.3 × 10^−7^–2 × 10^−6^	4.99 × 10^−5^–1.07 × 10^−4^	2.74 × 10^−6^–5.91 × 10^−6^
Cr	3	0.075	1 × 10^−4^–3.01 × 10^−4^	3.81 × 10^−5^–1.14 × 10^−4^	3.84 × 10^−4^–1.15 × 10^−3^	1.13 × 10^−4^–3.38 × 10^−4^	2.01 × 10^−4^–5.02 × 10^−4^	7.63 × 10^−5^–1.14 × 10^−4^	7.67 × 10^−4^–1.15 × 10^−3^	2.25 × 10^−4^–3.38 × 10^−4^
Ni	20	5.4	0–2.86 × 10^−4^	0–2.01 × 10^−5^	0–1.09 × 10^−3^	0–5.94 × 10^−5^	0–3.01 × 10^−5^	0–2.12 × 10^−6^	1.09 × 10^−4^–1.15 × 10^−3^	0–6.25 × 10^−6^
HI_ing/derm_	–	–	2.33 × 10^−4^–3.24 × 10^−3^	1.075 × 10^−4^–3.00 × 10^−4^	4.59 × 10^−3^–1.24 × 10^−2^	3.18 × 10^−4^–8.88 × 10^−4^	2.16 × 10^−3^–4.24 × 10^−3^	2.00 × 10^−4^–3.51 × 10^−4^	9.34 × 10^−3^–1.54 × 10^−2^	5.90 × 10^−4^–1.04 × 10^−3^

**Table 7:** Chronic risk assessment (CDI) for the metals in the groundwater samples from settlements around the Bosomtwe Crater Lake and water samples from the lake through the ingestion pathway during the wet and dry seasons for adults and children

	Dry season				Wet season			
	Lake water		Underground		Lake water		Groundwater	
	Adults	Children	Adult	Children	Adult	Children	Adult	Children
Fe	6.29 × 10^−3^–1.19 × 10^−2^	2.40 × 10^−2^–4.56 × 10^−2^	6.29 × 10^−3^–1.82 × 10^−2^	2.40 × 10^−2^–6.96 × 10^−2^	8.49 × 10^−3^–6.24 × 10^−2^	3.24 × 10^−2^–2.39 × 10^−1^	8.17 × 10^−3^–3.21 × 10^−2^	3.12 × 10^−2^–1.22 × 10^−1^
Pb	1.89 × 10^−3^–5.34 × 10^−3^	7.20 × 10^−3^–2.04 × 10^−2^	2.83 × 10^−3^–5.34 × 10^−3^	1.08 × 10^−2^–2.04 × 10^−2^	1.26 × 10^−3^–3.46 × 10^−3^	4.80 × 10^−3^–1.32 × 10^−2^	1.57 × 10^−3^–3.77 × 10^−3^	6.00 × 10^−3^–1.44 × 10^−2^
Zn	3.14 × 10^−3^–6.60 × 10^−3^	1.20 × 10^−2^–2.52 × 10^−2^	3.77 × 10^−3^–1.07 × 10^−4^	1.44 × 10^−2^–5.91 × 10^−6^	2.83 × 10^−3^–5.03 × 10^−5^	1.08 × 10^−2^–1.92 × 10^−2^	4.40 × 10^−3^–8.8 × 10^−3^	1.68 × 10^−2^–3.36 × 10^−2^
Cr	3.14 × 10^−4^–1.57 × 10^−3^	1.20 × 10^−3^–6.00 × 10^−3^	3.14 × 10^−4^–8.80 × 10^−^3	1.20 × 10^−3^–3.36 × 10^−3^	3.14 × 10^−4^–6.29 × 10^−4^	1.20 × 10^−3^–2.40 × 10^−3^	3.14 × 10^−4^–9.43 × 10^−4^	1.20 × 10^−3^–3.60 × 10^−3^
Ni	0–6.29 × 10^−4^	0–2.40 × 10^−3^	0–6.29 × 10^−4^	0–2.40 × 10^−3^	0–0	0–0	0–5.97E−03	0–2.28E−02

The carcinogenic risk (CR) assessment for the metals are through ingestion of both lake water and groundwater in the lake and its environs is given in Table 8. The CR value has been calculated for calculated for Cr and Pb only because the value of cancer slop for Zn, Fe and Ni could not be assessed in the Integrated Risk Information System (IRIS, provided by USEPA database; USEPA 2005). Generally a CR value greater than 1 in a million (10^−6^) is considered significant by USEPA. The results show that Pb and Cr exhibited ranges of carcinogenic indices exceeding 10^−6^ for both lake and groundwater for both adults and children for both seasons. This indicate that ingestion of both lake and groundwater from the lake and its environs poses carcinogenic risk with regard to the level of Pb and Cr. Hence appropriate control measures and interventions should be put in place to protect the health of the human population in the study area.

**Table 8:** Carcinogenic risk assessment (CR_ing_) for the metals in the groundwater from settlements around the Bosomtwe Crater Lake and water samples from the lake through the ingestion pathway during the wet and dry seasons for adults and children

	Dry season				Wet season			
	Lake water		Underground		Lake water		Groundwater	
	Adults	Children	Adult	Children	Adult	Children	Adult	children
Pb	2.13 × 10^−4^–6.03 × 10^−4^	8.12 × 10^−4^–2.30 × 10^−3^	3.19 × 10^−4^–6.03 × 10^−4^	1.22 × 10^−3^–2.30 × 10^−3^	1.42 × 10^−4^–3.90 × 10^−4^	5.41 × 10^−4^–1.49 × 10^−3^	1.77 × 10^−4^–4.25 × 10^−4^	6.77 × 10^−4^–1.62 × 10^−3^
Cr	6.03 × 10^−7^–3.01 × 10^−6^	2.3 × 10^−6^–1.15 × 10^−5^	6.03 × 10^−7^–1.81 × 10^−6^	2.3 × 10^−6^–6.9 × 10^−6^	6.03 × 10^−7^–1.21 × 10^−6^	2.3 × 10^−6^–4.6 × 10^−6^	6.03 × 10^−7^–1.81 × 10^−6^	2.3 × 10^−6^–6.9 × 10^−6^

## Conclusion

The mean levels of Fe and Pb were above the WHO values for both lake and groundwater for both wet and dry seasons whereas the mean levels of Zn and Cr were below the WHO values for both lake and groundwater for both seasons. The hazard quotients (HQ) and health hazard indices (HI) through ingestion and dermal contact of lake and groundwater in towns around the lake for both adults and children gave values which were below the acceptable limit (< 1), indicating the absence of non-carcinogenic health risk to the communities. The study however reveals that ingestion of both lake and groundwater from the lake and its surroundings poses carcinogenic risk with regard to the level of Pb and Cr. Hence appropriate control measures and interventions should be put in place to protect the health of the human population in the study area.

## Abbreviations

CDI: Chronic risk assessment
CR: Carcinogenic risk
WHO: World Health Organization
HQ: Hazard quotient
AAS: Atomic absorption spectrometer
HI: Hazard index
IRIS: Integrated Risk Information System
